# The organisation of spinoparabrachial neurons in the mouse

**DOI:** 10.1097/j.pain.0000000000000270

**Published:** 2015-06-22

**Authors:** Darren Cameron, Erika Polgár, Maria Gutierrez-Mecinas, Maria Gomez-Lima, Masahiko Watanabe, Andrew J. Todd

**Affiliations:** a Institute of Neuroscience and Psychology, College of Medical, Veterinary and Life Sciences, University of Glasgow, Glasgow, United Kingdom; b Department of Anatomy, Hokkaido University School of Medicine, Sapporo, Japan

**Keywords:** Spinal cord, Lamina I, Neurokinin 1 receptor, Giant cell, Lateral parabrachial area, Substance P

## Abstract

The anterolateral tract (ALT), which originates from neurons in lamina I and the deep dorsal horn, represents a major ascending output through which nociceptive information is transmitted to brain areas involved in pain perception. Although there is detailed quantitative information concerning the ALT in the rat, much less is known about this system in the mouse, which is increasingly being used for studies of spinal pain mechanisms because of the availability of genetically modified lines. The aim of this study was therefore to determine the extent to which information about the ALT in the rat can be extrapolated to the mouse. Our results suggest that as in the rat, most lamina I ALT projection neurons in the lumbar enlargement can be retrogradely labelled from the lateral parabrachial area, that the majority of these cells (∼90%) express the neurokinin 1 receptor (NK1r), and that these are larger than other NK1r-expressing neurons in this lamina. This means that many lamina I spinoparabrachial cells can be identified in NK1r-immunostained sections from animals that have not received retrograde tracer injections. However, we also observed certain species differences, in particular we found that many spinoparabrachial cells in laminae III and IV lack the NK1r, meaning that they cannot be identified based solely on the expression of this receptor. We also provide evidence that the majority of spinoparabrachial cells are glutamatergic and that some express substance P. These findings will be important for studies designed to unravel the complex neuronal circuitry that underlies spinal pain processing.

## Abstract 2

This study suggests that 5% of lamina I neurons are projection cells, which most express the neurokinin 1 receptor, and that these can generally be distinguished from interneurons based on their larger size.

## 1. Introduction

Nociceptive, thermal, and pruritic information is conveyed from spinal cord to brain through the anterolateral tract (ALT).^50^ Cells of origin of the ALT are concentrated in lamina I and scattered throughout the deeper laminae (III-VI). Their supraspinal targets include thalamus, periaqueductal grey (PAG) matter, lateral parabrachial area (LPb), and certain medullary nuclei. Individual ALT neurons can send axon collaterals to several of these regions.^2,22,30,41,47^

Quantitative studies in rat lumbar enlargement have demonstrated that ALT projection cells account for ∼5% of lamina I neurons. Virtually all of these can be retrogradely labelled from LPb, and they can therefore be classified as spinoparabrachial neurons.^2,41,47^ Studies in rat suggest that the majority of lamina I spinoparabrachial neurons respond to noxious stimuli.^5,7^ The neurokinin 1 receptor (NK1r), which is present at high levels in lamina I,^8,10,25,55^ is expressed by ∼70 to 80% of lamina I ALT neurons in rat^4,15,50^ and by a population of laminae III and IV projection neurons with dorsal dendrites that arborise in the superficial laminae.^32,52^ NK1r-expressing projection neurons in laminae I, III, and IV are densely innervated by nociceptive primary afferents that contain substance P (SP).^24,32,53^ Ablation of NK1r^+^ neurons by intrathecal injection of SP-saporin conjugate results in dramatic reduction of hyperalgesia in chronic pain states,^28,34^ which is thought to result from destruction of NK1r-expressing ALT neurons.^50^ Although the NK1r is also expressed by excitatory interneurons in lamina I,^25^ these are significantly smaller than the projection cells.^3,11^ This means that NK1r-expressing projection neurons in rat can generally be identified based on size, thus avoiding the need for retrograde tracing experiments in many cases. We have also identified a population of giant lamina I projection cells that generally lack the NK1r and are characterised by the high density of excitatory and inhibitory synapses on their cell bodies and dendritic trees.^35,43^

The mouse is increasingly used for investigations of spinal pain mechanisms, but little is known about the ALT in this species.^14^ This study therefore compared the organisation of the mouse spinoparabrachial projection with that of the rat. We aimed to determine the number of lamina I spinoparabrachial neurons, the proportion that expressed NK1r, and whether, as in rat,^3^ these could be distinguished from other NK1r^+^ neurons based on soma size. We also tested whether giant lamina I cells in mouse belong to the spinoparabrachial tract. In preliminary studies, we found that NK1r-immunoreactive cells were infrequent in laminae III and IV of the mouse; therefore, we tested whether spinoparabrachial neurons were present in these laminae. We have shown that the somatostatin receptor sst_2A_ is virtually restricted to GABA-immunoreactive (inhibitory) neurons in laminae I and II^37,54^; however, Gamboa-Esteves et al.^18^ detected sst_2A_ on some lamina I projection neurons in rat. We therefore tested for sst_2A_ expression by lamina I spinoparabrachial neurons and used anterograde tracing to assess the whether any of these cells were GABAergic. Finally, we looked for expression of neuropeptides, which has been reported for spinoparabrachial cells in other species.^9,48^

## 2. Methods

### 2.1. Animals

All experiments were approved by the Animal Welfare and Ethical Review Board of the University of Glasgow and were performed in accordance with the UK Animals (Scientific Procedures) Act 1986.

Six adult C57Bl/6 mice of either sex (22-35 g; Biological Services, University of Glasgow) were used in this study. They were anaesthetised with isoflurane and placed in a stereotaxic or spinal frame, after which anaesthetic was administered through a mask attached to the frame. In 4 cases, a burr hole was made through the skull, and a single injection of 200 or 300 nl of 1% cholera toxin B subunit (CTb) was targeted on the left LPb. In the other 2 cases, the lumbar vertebral column was exposed, and 2 injections of 150 nl of 1% CTb were made into the dorsal horn on one side of the spinal cord at a depth of 300 μm below the pial surface, through the intervertebral spaces rostral and caudal to the T13 vertebra. In all cases, injections were made through a glass micropipette, which was left in place for 5 minutes after completion of the injection, to minimise leakage of tracer back up the track. All animals made an uneventful recovery from anaesthesia. After a 4- or 5-day survival period, they were reanaesthetised with pentobarbitone (20 mg, intraperitoneally) and perfused through the heart with a fixative that contained 4% freshly depolymerised formaldehyde.

The brains and lumbar spinal cords were removed from all animals and postfixed for at least 4 hours, before being cut into sections with a vibrating blade microtome.

### 2.2. General features of tissue processing and analysis

From all 6 mice, the region of brain or spinal cord containing the injection site was cut into transverse sections (100 μm for brain, 60 μm for spinal cord), and injection sites were revealed by incubating the sections in goat antibody against CTb at 1:50,000 to 200,000 and performing an immunoperoxidase reaction, as described previously.^1,47^

The L4 spinal cord segments from the LPb-injected animals were cut into 2 series of 60-μm-thick transverse sections (each consisting of alternating sections), while the L5 segment and either L2 or L3 were cut into 60 μm horizontal sections. The part of the brainstem that included the LPb from the spinal-injected mice was cut into 4 series of 60 μm tranverse (coronal) sections. The sections were reacted for immunofluorescence staining, by incubating them for 3 days in mixtures of primary antibodies and then in mixtures of species-specific secondary antibodies that were raised in donkey. The secondary antibodies were conjugated to Alexa 488, Alexa 647, Rhodamine Red, Pacific Blue, or biotin (Jackson ImmunoResearch, West Grove, PA) and were used at 1:500, apart from those conjugated to Pacific Blue (1:200) or Rhodamine Red (1:100). Biotinylated secondary antibodies were revealed with avidin–Pacific Blue (1:1000; Life Technologies, Paisley, United Kingdom). After immunoreaction, the sections were mounted in antifade medium and stored at −20°C. All antibodies were diluted in phosphate-buffered saline that contained 0.3% Triton X-100 and 5% normal donkey serum. The sources and dilutions of the primary antibodies are shown in Table 1.

**Table 1:**
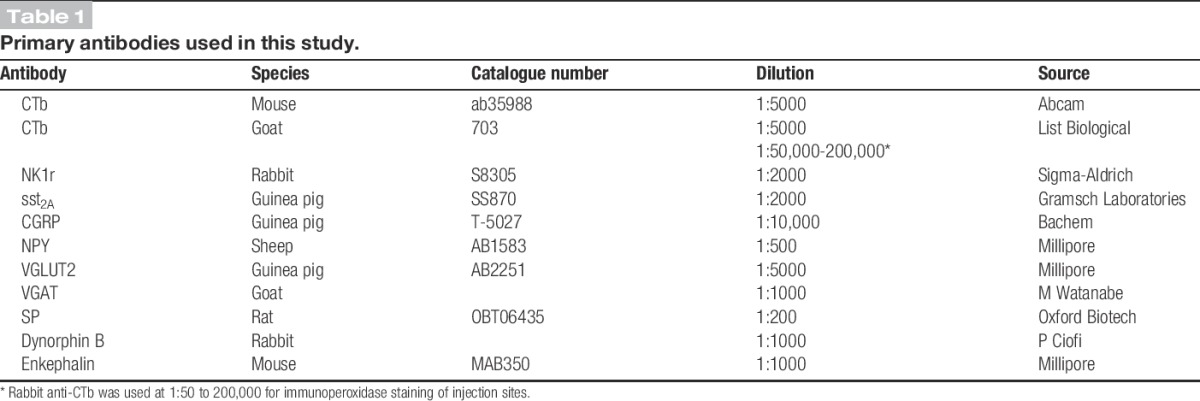
Primary antibodies used in this study.

Sections reacted for immunofluorescence were scanned with a Zeiss LSM 710 confocal microscope equipped with Argon multiline, 405 nm diode, 561 nm solid state and 633 nm HeNe lasers, and a spectral detection system. Confocal image stacks were acquired by scanning through a 40× or 63× oil immersion lens (numerical aperture 1.3 or 1.4, respectively) with the aperture set to 1 Airy unit. Scans through the spinal cord were scanned at a z-spacing of 1 or 2 μm, whereas those from the LPb were scanned at 0.5 μm z-spacing.

### 2.3. Quantification of CTb-labelled lamina I neurons and expression of NK1 and sst_2A_ receptors

One series of transverse sections from the L4 segment of the mice that had received injections into the LPb was reacted with antibodies against CTb (raised in mouse), NK1r, and sst_2A_. Seven sections were selected from each animal, before the CTb-immunoreactivity was viewed. Two overlapping fields were scanned to include the whole superficial dorsal horn through the full thickness of the section, and these scans were analysed with Neurolucida for Confocal software (MBF Bioscience, Williston, VT). The outline of the dorsal horn contralateral to the LPb injection was initially drawn, together with the ventral border of lamina I, which could be identified due to the plexus of NK1r-immunoreactive dendrites within the lamina. The channel representing CTb was visualised, and the locations of CTb-labelled cells were plotted onto these drawings. To avoid overcounting cells that were split by sectioning,^20^ we included cells if at least part of the nucleus (revealed as a filling defect in the CTb) was present in the first optical section in the z-series and excluded them if part of the nucleus was present in the last optical section.^1,41,47^ Because the analysis was performed on an alternate series of sections, it would not be possible for the same cell to be counted a second time on another section. The NK1r and sst_2A_ channels were then viewed, and the presence or absence of each of these types of immunoreactivity was noted for each of the CTb-labelled cells in the sample. In addition, a search was made for all lamina I cells that were immunoreactive for both NK1r and sst_2A_, and these were then examined to see whether they were CTb-labelled.

### 2.4. Analysis of soma sizes for NK1r neurons and identification of giant cells

Horizontal sections from 2 segments (L5 and either L2 or L3) of each of the 4 LPb-injected mice were reacted with antibodies against CTb (mouse), NK1r, VGLUT2, and VGAT.

The L5 sections were used to assess the soma sizes of NK1r-immunoreactive lamina I neurons.^3^ The sections that contained numerous retrogradely labelled lamina I neurons on the side contralateral to the LPb injection were initially identified, and in this way, either 1 or 2 sections were selected from each animal. These were then scanned through the 40× lens with the confocal microscope to generate overlapping z-stacks (2 μm z-separation) that included the whole of lamina I apart from its lateral part, which was not analysed due to the differing orientation of cells in this region.^3^ The scans were analysed with Neurolucida for Confocal, such that only the channel representing NK1r-immunoreactivity was initially visible. The ventral border of lamina I could be readily identified because the density of NK1r-immunoreactive cells is very low in lamina II. All NK1r^+^ cells with a soma that was entirely contained within the section were identified and drawn, and the cross-sectional area of the soma in the horizontal plane was measured from the drawings.^3,43^ The channel representing CTb was then viewed, and the presence or absence of retrogradely transported CTb was noted for each of the NK1r^+^ cells.

Sections from the 2 segments that had been cut into horizontal sections (L5 and either L2 or L3) were examined to look for giant lamina I cells, which can be recognised by the high density of VGLUT2- and VGAT-immunoreactive boutons that outline their cell bodies and dendrites.^35,43^ These were initially identified by viewing the rhodamine channel (corresponding to VGLUT2) and then confirming the presence of numerous VGAT-immunoreactive boutons. Confocal z-stacks were then scanned to include the soma and proximal dendrites, and the presence or absence of CTb and NK1r was noted for each cell.

### 2.5. Projection neurons in laminae III and IV

Retrogradely labelled neurons in laminae III and IV were examined in the second series of transverse sections of L4 from the 4 mice that received LPb injections. These sections were reacted with antibodies against CTb (mouse antibody), NK1r, calcitonin gene-related peptide (CGRP), and neuropeptide Y (NPY). This reaction was performed because the NK1r-immunoreactive ALT cells in laminae III and IV of the rat spinal cord are densely innervated by both peptidergic (CGRP^+^) primary afferents^32^ and NPY-containing inhibitory interneurons.^39,40^ The sections were initially viewed with fluorescence microscopy, and those that contained CTb-labelled cells in laminae III and IV on the side contralateral to the LPb injection were identified. These cells were then scanned with the confocal microsope through the 40× lens to generate z-stacks (2 μm z-separation) through the cell body and as much of the dendritic tree as was visible. In each case, the presence or absence of NK1r-immunoreactivity was noted, and we determined whether the cell body and dendrites were associated with clusters of CGRP- and/or NPY-immunoreactive axons.

### 2.6. Anterograde tracing

One series of sections through the brainstem of the 2 mice that received spinal injections of CTb was reacted with antibodies against CTb (mouse antibody), VGAT, and VGLUT2. A single section through the LPb that contained numerous CTb-labelled boutons was selected for analysis and scanned with an oil immersion lens to produce z-series of 25 or 30 optical sections at 0.5 μm z-spacing. Overlapping scans were obtained from the entire region that contained CTb-labelled boutons in this section. The resulting z-stacks were viewed with Neurolucida for Confocal, and a 5 × 5 μm grid was superimposed on the confocal images. A single optical section within the z-stack was chosen before VGAT or VGLUT2 immunostaining were viewed, and CTb-labelled boutons were sampled by selecting the bouton nearest the bottom left hand corner of successive grid squares, starting near the dorsal- and lateral-most part of the LPb that contained these boutons and progressing through the grid squares in a dorsal-ventral and then lateral–medial direction until the entire region containing CTb boutons had been covered.^45^ The numbers of boutons analysed in the 2 mice were 100 and 208.

Another series of sections was used to investigate neuropeptide expression by anterogradely labelled terminals in the LPb. The sections were reacted with antibodies against CTb (goat antibody), enkephalin, dynorphin B, and SP. From each animal, 2 sections that contained numerous CTb-labelled boutons were selected and scanned as described above. Again, CTb^+^ boutons were selected before the other types of immunostaining were viewed, and the selected boutons were then examined to look for neuropeptide immunoreactivity. To compensate for any bias towards larger profiles in the selection process,^20^ we measured the z-axis lengths of each of the selected boutons by determining the number of z-sections on which it was present.^21,45^ The numbers of boutons analysed in this part of the study were 280 and 391 for the 2 animals.

### 2.7. Antibody characterisation

Specificity of the CTb antibodies is demonstrated by the lack of staining in regions that did not contain injected or transported tracer. The NK1r antibody, raised against amino acids 393 to 407 of the rat NK1r, recognises a 46 kDa band in Western blots of rat brain extracts, and it has been shown that there is no staining with this antibody in mice in which the NK1r has been deleted.^42^ The sst_2A_ antibody was raised against the C-terminal 15 amino acids of the mouse receptor, conjugated to keyhole limpet haemocyanin, and staining is abolished by pre-incubation of this peptide (manufacturer's specification). The CGRP antibody detects both α and β forms of the peptide. The NPY antibody was raised against synthetic NPY conjugated to bovine thyroglobulin and is reported to show negligible cross-reactivity with a range of other peptides, including enkephalins, somatostatin, SP, angiotensin, or vasoactive intestinal polypeptide (manufacturer's specification). The VGLUT2 antibody was raised against a synthetic peptide from rat VGLUT2, and we have shown that it stains identical structures to a well-characterised rabbit anti-VGLUT2.^51^ The VGAT antibody was raised against amino acids 31 to 112 of the mouse protein and recognises a single band of the appropriate size on Western blots.^31^ The monoclonal SP antibody detects the C-terminal 5 to 8 amino acids of the peptide^10^ and does not appear to recognise neurokinin B.^29,38^ The dynorphin B antibody was raised against the full peptide and does not cross-react with enkephalins.^19^ Staining with this antibody is absent in the spinal cord of preprodynorphin knockout mice.^23^ The monoclonal antibody against enkephalin recognises both Met- and Leu-enkephalin and shows significant cross-reactivity with C-terminal extended Met-enkephalin hexapeptides and heptapeptides, but not with either beta-endorphin or dynorphin (manufacturer's specification).

### 2.8. Statistics

The Mann–Whitney *U* test was used to compare the soma areas of retrogradely labelled and nonlabelled NK1r-immunoreactive lamina I neurons. Differences in the z-axis lengths of CTb-labelled boutons in the LPb that were tested for neuropeptide immunoreactivity were compared using a *t* test.

## 3. Results

### 3.1. Injection sites

**Figure 1:**
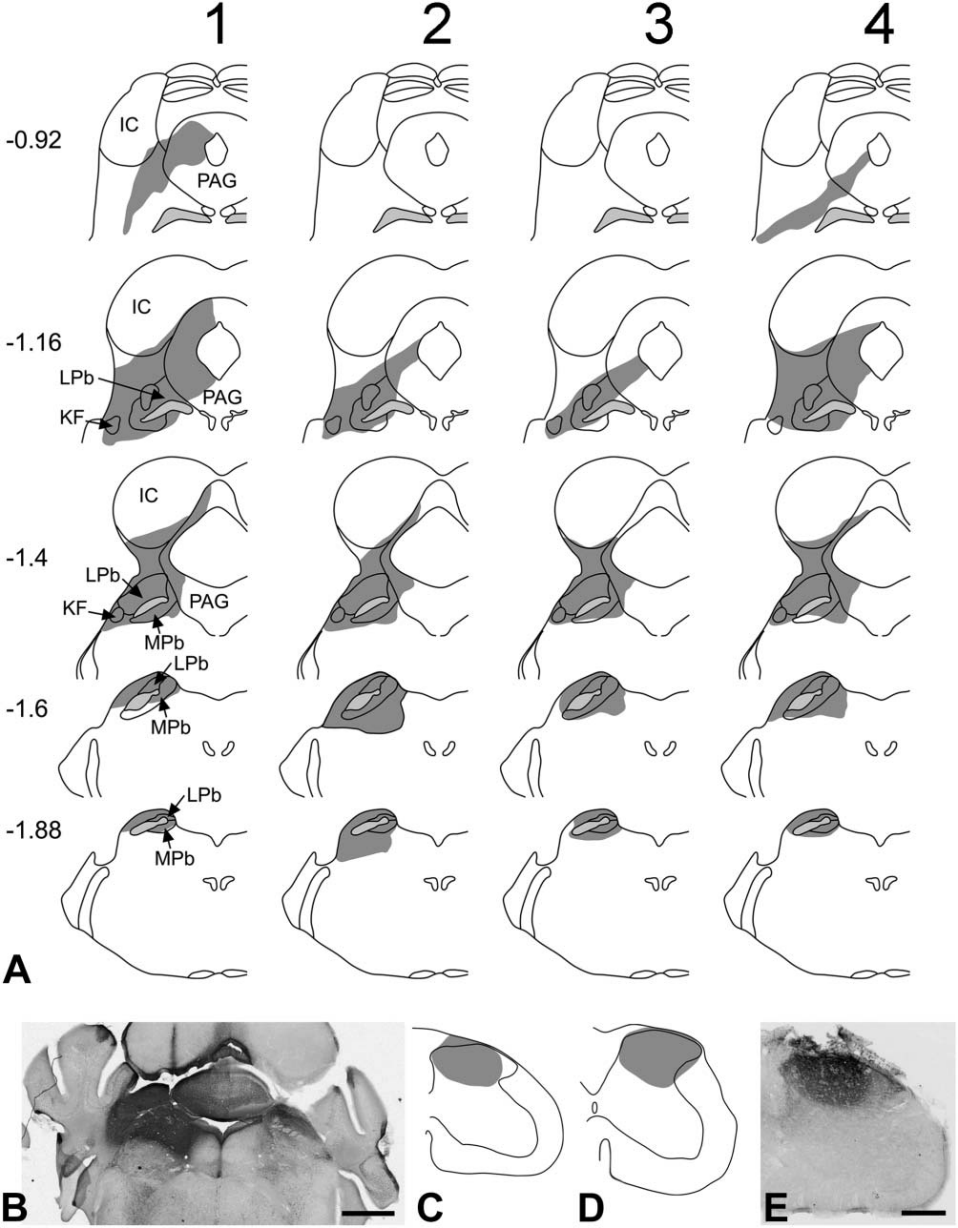
Injection sites in the 6 mice used in this study. (A) Injection sites in the 4 experiments in which CTb was injected into the lateral parabrachial area. Each vertical column represents a single experiment. The darker shaded areas show the spread of tracer, while the pale shaded area is the superior cerebellar peduncle. Numbers to the left of the drawings correspond to the position of the section posterior to the interaural plane. Drawings are based on those of Franklin and Paxinos.^16^ (B) A section through the brain from the mouse #4, corresponding to a level of ∼1.5 mm caudal to the interaural plane. The CTb injection site appears as a dark area, and near the top of the image, the pipette track can be seen passing through the inferior colliculus. (C and D) Drawings indicating the core of the injection site in the 2 mice that received intraspinal injections of CTb. (E) A section from the spinal cord of the mouse represented in (C). Scale bars: (B), 1 mm; (C), 250 μm. IC, inferior colliculus; KF, Kölliker-Fuse nucleus; LPb, lateral parabrachial area; MPb, medial parabrachial area; PAG, periaqueductal grey.

Injection sites in the 6 mice are illustrated in Figure 1. In all 4 mice that received CTb injections into the brainstem, the immunoperoxidase reaction product filled the entire LPb, which extends from 1.16 to 1.88 mm caudal to the interaural plane^17^ (Fig. 1A and B). The injection sites also included most of the medial parabrachial area and Kolliker-Fuse nucleus and the caudal-most part of the PAG in all cases. There was variable spread into surrounding areas, including the cuneiform nucleus and cerebellum. The spinal cord injections were located in the L3 and L4 segments, and in both cases, these filled most of laminae I-III of the ipsilateral dorsal horn (Fig. 1C-E) at the levels of the injection.

### 3.2. Lamina I spinoparabrachial neurons: quantification and expression of NK1r and sst_2A_ receptor

Altogether, 276 CTb^+^ lamina I neurons (range, 66-75; mean, 69) were identified in the transverse sections of the L4 segments that were analysed from the 4 LPb-injected mice (7 sections per mouse; Table 2). This corresponds to a mean of 9.86 cells in each 60-μm-thick section, and as we have estimated that the length of the L4 segment in the mouse is 1.45 mm,^37^ this suggests that there would have been an average of 238 spinoparabrachial lamina I cells on the contralateral side in the entire L4 segment in these animals. There are ∼4500 lamina I neurons on each side in this segment in the mouse,^37^ and so we estimate that approximately 5.3% of these cells belong to the spinoparabrachial tract.

**Table 2:**
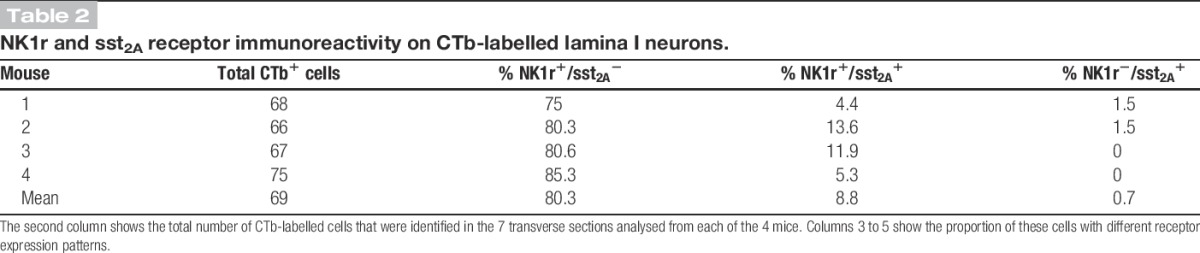
NK1r and sst_2A_ receptor immunoreactivity on CTb-labelled lamina I neurons.

**Figure 2:**
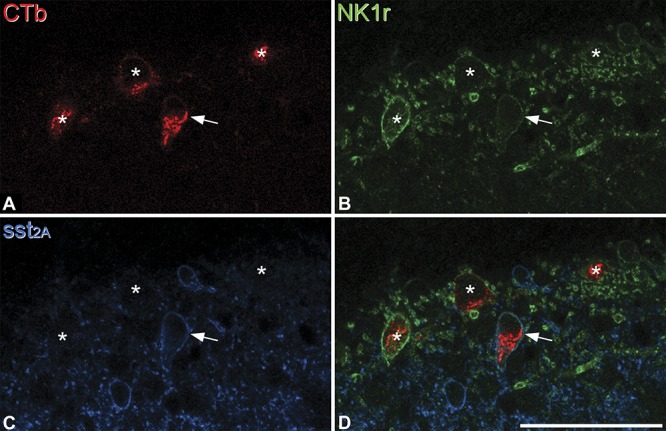
Expression of NK1r and sst_2A_ by retrogradely labelled spinoparabrachial neurons in lamina I. Confocal images showing a single optical section through the superficial dorsal horn in the L4 segment, contralateral to the lateral parabrachial area injection site. (A) CTb labelling (red) in 4 lamina I neurons identifies these as belonging to the spinoparabrachial tract. (B-D) Immunostaining of the section for the NK1r (green) and sst_2A_ (blue) shows that one of these cells (marked with an arrow) expresses both receptors, while the other 3 (asterisks) are only NK1r-immunoreactive. Scale bar: 20 μm.

As reported previously, immunoreactivity for both NK1r and sst_2A_ receptor was present on cell bodies and dendrites in lamina I, and these were found on largely separate neuronal populations, although a few cells were immunoreactive for both receptors.^37^ The majority of the CTb-labelled lamina I cells were NK1r-immunoreactive (Fig. 2), with the proportion varying between 79.4% and 93.9% among the 4 mice (mean, 89.1%) (Table 2). Sst_2A_-immunoreactivity was found on 26 of the CTb-labelled cells (between 4 and 10 per mouse), and this corresponded to between 5.3% and 15.1% (mean, 9.5%) of the retrogradely labelled neurons (Table 2). All but 2 of the sst_2A_-immunoreactive CTb-labelled lamina I cells were also NK1r^+^. When the NK1r and sst_2A_ channels were viewed, it was found that all of the lamina I cells that were immunoreactive for both receptors were retrogradely labelled with CTb.

### 3.3. Soma sizes of NK1r^+^ lamina I neurons

**Figure 3:**
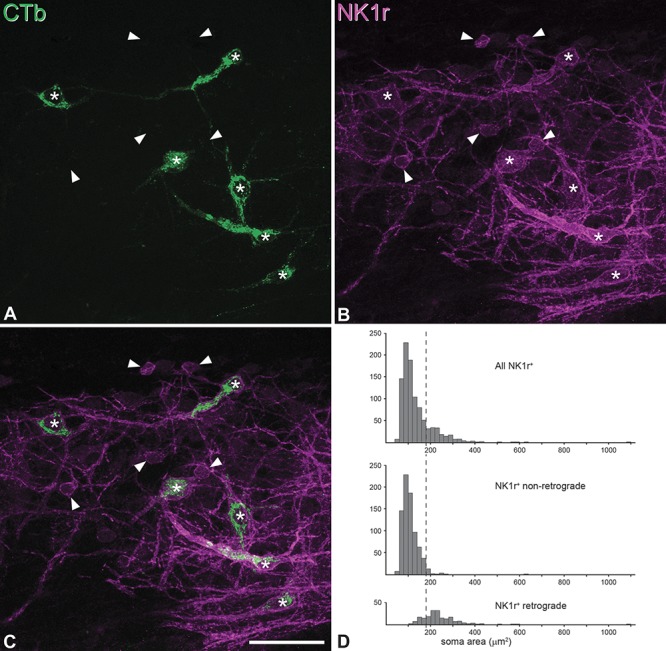
NK1r-immunoreactive projection neurons in lamina I. (A-C) Confocal images from a horizontal section through the L5 segment of one of the mice that had received an injection of CTb into the lateral parabrachial area. The section has been scanned to reveal CTb (green) and NK1r (magenta). Six retrogradely labelled (CTb-positive) neurons are indicated with asterisks, and each of these is NK1r-immunoreactive. In addition, several smaller NK1r-immunoreactive cells that are not retrogradely labelled (CTb-negative) are visible, and 5 of these are marked with arrowheads. Projection from 5 optical sections at 2 μm z-spacing. Scale bar: 50 μm. (D): Histograms showing the numbers of lamina I NK1r-immunoreactive cells with different soma areas. The top graph shows the results for all cells (All NK1r^+^), while the middle and lower graphs show those that were not labelled (NK1r^+^ nonretrograde) or retrogradely labelled (NK1r^+^ retrograde), respectively. The dashed line corresponds to a soma area of 180 μm^2^. Note that most of the nonretrogradely labelled neurons are smaller than this, whereas most retrogradely labelled cells are larger.

Between 187 and 279 (mean, 245.8) NK1r-immunoreactive lamina I cells were identified and analysed in the horizontal sections through the contralateral sides of the L5 segments of the 4 LPb-injected mice, and an example of the immunostaining for NK1r and CTb is shown in Figure 3A-C. The soma cross-sectional areas of the NK1r^+^ cells ranged from 52 to 1084 μm^2^ and showed a bimodal distribution, with a major peak between 50 and 180 μm^2^, a smaller peak between 180 and 400 μm^2^, and a few cells with larger areas (Fig. 3D). Between 17.3% and 25.2% (mean, 20.2%) of the NK1r-immunoreactive neurons were retrogradely labelled with CTb. There was a clear difference in the soma areas of the NK1r cells that were retrogradely labelled with CTb (range, 116-1084 μm^2^; median, 228 μm^2^) and those that were not (range, 52-625 μm^2^; median, 101 μm^2^), and this was highly significant (*P* < 0.0001; Mann–Whitney *U* test). The majority (763/785, 97%) of NK1r cells with soma areas <180 μm^2^ were not retrogradely labelled with CTb, whereas most (158/198, 80%) retrogradely labelled neurons had soma areas >180 μm^2^. If a soma area of 200 μm^2^ was taken as a cutoff value, then 140 (71%) of the retrogradely labelled cells would be included, but only 8 (1%) of the nonretrogradely labelled cells. In other words, 95% of the NK1r cells with soma areas ≥200 μm^2^ were retrogradely labelled from the LPb in these experiments.

### 3.4. Giant cells

**Figure 4:**
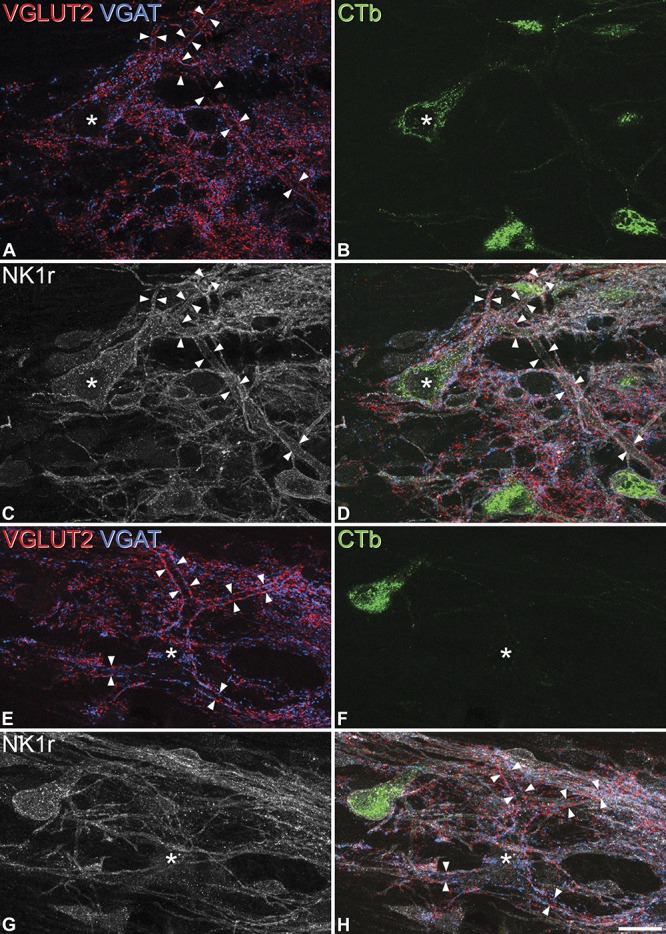
Giant lamina I neurons seen in horizontal sections. (A-D): Confocal images showing a giant cell that was retrogradely labelled with CTb (green) and immunoreactive for the NK1r (grey). Note that many boutons containing VLGUT2 (red) or VGAT (blue) are associated with the cell body (*) and that these surround the dendrites (indicated by arrowheads). (E-H): Another giant cell that was not retrogradely labelled and lacked NK1r-immunoreactivity. Again, the soma (*) and dendrites (between arrowheads) are associated with numerous VGLUT2 and VGAT boutons. The 2 sets of images are projections of 5 (A-D) and 6 (E-H) optical sections at 1 μm z-spacing. Scale bar: 20 μm.

Altogether, 25 giant cells were identified in the horizontal sections from the 4 LPb-injected mice (3-8 cells per animal). In each case, the cell received numerous contacts from both VGLUT2- and VGAT-immunoreactive boutons. These boutons outlined the dendritic tree and were also numerous on the cell body (Fig. 4A and E). However, unlike the situation in the rat, in which virtually all of the giant cells can be retrogradely labelled from the LPb,^43^ only 8 of these cells (32%, between 0 and 3 in each mouse) were labelled with CTb (Fig. 4). As in the rat, we found that most of the giant cells (16/25, 64%) lacked the NK1r, although interestingly, all of the CTb-labelled cells (together with one of the cells that lacked CTb) were NK1r-immunoreactive (Fig. 4).

### 3.5. Spinoparabrachial neurons in laminae III and IV

**Figure 5:**
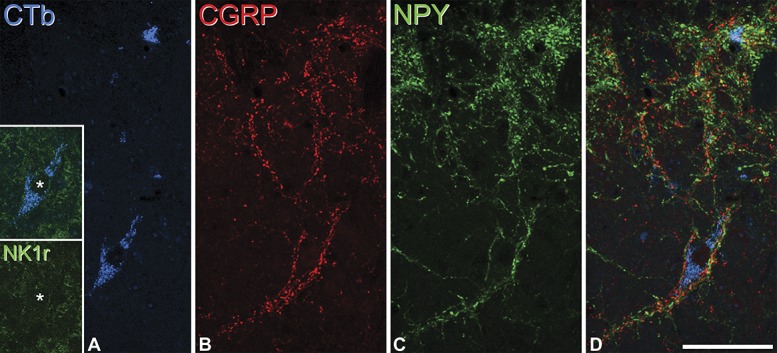
Innervation of a lamina III NK1r-negative projection neuron by CGRP- and NPY-containing axons. (A): Confocal image showing part of a transverse section through a lamina III neuron that was retrogradely labelled with CTb (blue). (B-D): The cell body is surrounded by axons that contain CGRP (red) or NPY (green). The inset in (A) shows the region through the cell body (*) stained for the NK1r, and this cell is not immunoreactive for the receptor. The images are projections of 4 optical sections at 2 μm z-spacing. Scale bar: 50 μm. CGRP, calcitonin gene-related peptide; NPY, neuropeptide Y.

Retrogradely labelled neurons in laminae III and IV were counted in transverse sections of the L4 segment from each of the 4 mice that had received an injection of CTb into the LPb. This analysis was performed on sections that had been reacted with antibodies against CTb, NK1r, CGRP, and NPY, and between 11 and 13 sections were examined for each mouse (Table 3). Altogether, 18 CTb-labelled cells (range, 3-6; mean, 4.5) were found in the contralateral dorsal horn in these laminae. Eight of these cells were NK1r-immunoreactive (Table 3), but these generally showed only weak or moderate immunoreactivity. Because of the small numbers found in each animal, we calculated the mean proportion that were NK1r-immunoreactive (8/18, 44%) for the entire sample. All 18 of these cells were associated with dense clusters of both CGRP-immunoreactive and NPY-immunoreactive boutons, which contacted their cell bodies, as well as their dendrites when these were visible (Fig. 5). The total number of laminae III and IV spinoparabrachial cells in the contralateral part of the L4 segment was estimated by dividing the rostrocaudal length of the spinal cord examined (number of sections × 60 μm) by the total length of the segment (1.45 mm)^37^ and multiplying this by the number of cells identified in the sections analysed. This gave a mean value of ∼9 cells in the segment on the side contralateral to the LPb injection.

**Table 3:**
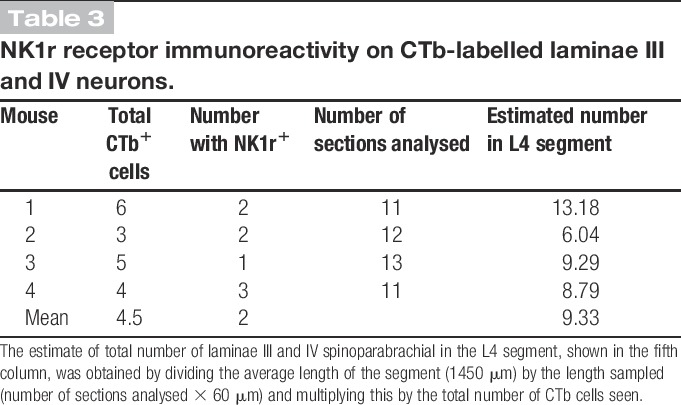
NK1r receptor immunoreactivity on CTb-labelled laminae III and IV neurons.

During the course of this analysis, we also observed a few NK1r-immunoreactive cells in this region that were associated with CGRP and NPY axons, but were not CTb-labelled, as well as occasional clusters of CGRP and NPY axons that were not associated with cells that were either NK1r^+^ or CTb-labelled.

### 3.6. Anterograde tracing

**Figure 6:**
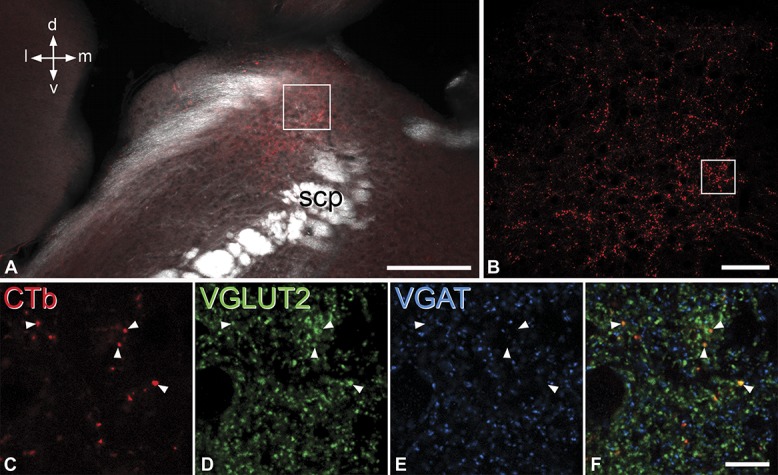
VGLUT2 expression by anterogradely labelled spinoparabrachial axon terminals. (A) Low-magnification image through the lateral parabrachial area (LPb) scanned to reveal CTb (red) and with dark-field optics. The LPb lies dorsal (d) and lateral (l) to the superior cerebellar peduncle (scp). (B): The region shown in the box in (A) scanned at high magnification (15 optical sections at 0.5 μm z-spacing) shows numerous CTb-labelled boutons. (C-F): Single optical section corresponding to the region shown in the box in (B), scanned to reveal CTb, VGLUT2 (green), and VGAT (blue). Four CTb-labelled boutons are indicated with arrowheads. These are all VGLUT2-immunoreactive and lack VGAT. Scale bars: (A), 200 μm; (B), 50 μm; (C-F), 10 μm.

After injection of CTb into the lumbar dorsal horn, CTb-immunoreactive boutons were present in large numbers in the LPb (Fig. 6A and B), in particular in the superior, central, dorsal, and internal subnuclei.^17^ However, little or no anterograde labelling was seen in the external or ventral parts of the LPb, or in the Köliker-Fuse nucleus or the medial parabrachial area. This distribution is generally similar to that reported in the corresponding regions of the rat LPb after injection of anterograde tracers into the lumbar spinal dorsal horn.^16,46^

The majority of the CTb-labelled boutons identified in the sections reacted for VGLUT2 and VGAT (95% and 99% in the 2 mice, mean, 97%) were VGLUT2-immunoreactive (Fig. 6C-F), whereas only 3% and 0.5% (mean 2%) were VGAT-immunoreactive. The remainder (2% and 1%; mean, 1.5%) were not immunoreactive for either transporter.

**Figure 7:**
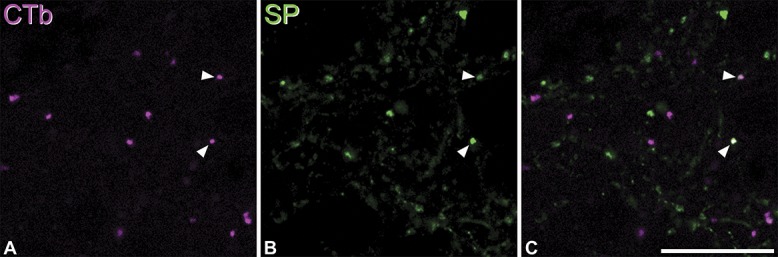
Expression of SP by anterogradely labelled spinoparabrachial terminals. (A and B): A single optical section through the lateral parabrachial area reacted to reveal CTb (magenta) and substance P (SP, green). (C): A merged image. Several CTb-labelled axonal boutons are visible, and some of these are SP-immunoreactive (2 indicated with arrowheads). Scale bar: 10 μm.

The results of peptide analysis are shown in Table 4, and an example of the immunostaining is shown in Fig. 7. Although the majority of CTb-labelled boutons did not show any type of peptide immunoreactivity, 16% were SP-immunoreactive, with much smaller proportions (1%-2%) showing dynorphin- or enkephalin-immunoreactivity. Very few of the CTb-labelled boutons showed more than one type of peptide immunoreactivity; altogether, we found 3 CTb-labelled boutons that were immunoreactive for SP and dynorphin and 1 that was immunoreactive for SP and enkephalin.

**Table 4:**
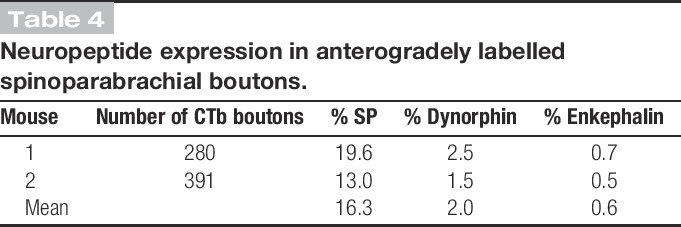
Neuropeptide expression in anterogradely labelled spinoparabrachial boutons.

## 4. Discussion

The main findings of this study are that (1) spinoparabrachial cells account for ∼5% of lamina I neurons in mouse lumbar enlargement; (2) ∼90% of these cells are NK1r^+^, and they are generally larger than other NK1r^+^ neurons in this lamina; (3) only a minority of giant lamina I cells are labelled from the LPb; (4) many laminae III and IV spinoparabrachial neurons lack the NK1r, but as in the rat, they are innervated by both CGRP- and NPY-containing axons; and (5) spinoparabrachial axons originating from the superficial dorsal horn are nearly all glutamatergic, with some expressing SP.

### 4.1. Identification of lamina I ALT neurons in the mouse

By injecting different tracers into LPb and the other known major targets of lamina I cells, we have shown that in the rat, virtually all lamina I ALT neurons can be retrogradely labelled from the contralateral LPb.^2,41,47^ We also demonstrated that among NK1r-immunoreactive neurons in this lamina, the majority of the large cells could be identified as spinoparabrachial neurons, without the need for retrograde tracing experiments.^3^ This raises 3 questions concerning this study: (1) did we label all lamina I spinoparabrachial neurons? (2) can most lamina I projection neurons be retrogradely labelled from an injection centred on LPb? and (3) can soma size be used to distinguish between projection and nonprojection cells among those that are NK1r-immunoreactive?

The LPb injection sites in our experiments included those regions that receive spinoparabrachial inputs,^16,46^ and numbers of retrogradely labelled lamina I were highly consistent across the 4 mice. Wang et al^56^ reported that very large injections of Fluoro-Gold (500 nl of 4% Fluoro-Gold) into the LPb in mice resulted in ∼100 retrogradely labelled lamina I neurons per 500 μm of lumbar spinal cord, which is somewhat higher than our estimate of 9.86 cells per 60 μm (equivalent to 82 cells per 500 μm). However, Wang et al included cells both contralateral and ipsilateral to the LPb injection, which probably accounts for the slightly higher cell counts that they obtained. In fact, we have shown that if 2 different tracers are injected into the right and left LPb in the rat, most lamina I neurons labelled from the ipsilateral LPb are also labelled from the contralateral side, indicating that lamina I spinoparabrachial neurons project either contralaterally or bilaterally.^47^ It is therefore likely that we labelled virtually all lamina I spinoparabrachial neurons in this study. As our aim was to label the maximum number of ALT neurons, we used relatively large injections of CTb that extended into adjacent regions including the caudal part of the PAG, which also receives collaterals from lamina I ALT neurons.^50^ More restricted injections would be needed to assess the proportion of these cells that actually project to LPb.

To test whether, as in the rat, most lamina I ALT cells are labelled from an injection into the LPb, it would be necessary to combine this with injections of a different tracer into all other known targets of these cells.^2,41,47^ However, our finding that the majority of large NK1r^+^ lamina I neurons (soma area >200 μm^2^) were retrogradely labelled strongly suggests that this is also true for the mouse. The alternative explanation would be that there was a significant population of ALT projection neurons that did not send collaterals to LPb and that this consisted mainly of small NK1r-expressing neurons or cells that lacked the receptor. Another indirect piece of evidence in support of this hypothesis is that the LPb injections labelled ∼5% of contralateral lamina I neurons, which is very similar to the proportion of neurons in this lamina that belong to the ALT in the rat.^47^

The majority of lamina I spinoparabrachial cells (∼90%) expressed the NK1r, and this proportion was even higher than that seen in the rat, in which 70% to 80% of spinoparabrachial neurons in this lamina are NK1r-immunoreactive.^2,4,15,47,52^ As in the rat,^3^ there was a highly significant size difference between retrogradely labelled and nonlabelled cells. For example, 95% of NK1r^+^ cells with soma areas >200 μm^2^ were CTb-labelled in the present series of experiments. This means that although immunostaining for the NK1r does not allow unequivocal identification of all lamina I projection neurons, it can be used to identify the larger NK1r-expressing spinoparabrachial cells with a reasonable degree of confidence and without the need for retrograde tracing experiments.

### 4.2. Differences between mouse and rat

We found 2 major species differences in the organisation of projection neurons. First, the proportion of lamina I giant cells that could be labelled from the LPb and second the lack of NK1r on many laminae III and IV spinoparabrachial neurons in the mouse.

Giant lamina I cells in the rat form a sparse, but distinctive, population that differ from NK1r-expressing projection neurons in their synaptic inputs and responses to noxious stimuli. They can readily be identified by the very high density of VGLUT2^+^ and VGAT^+^ boutons that almost completely outline their cell bodies and dendrites.^35^ They also differ from the NK1r^+^ cells in that they receive very few contacts from peptidergic primary afferents and show significantly less expression of the transcription factor Fos after noxious thermal stimulation.^35^ In the rat, we found that nearly all of these cells were retrogradely labelled from the LPb and that they account for ∼3% of spinoparabrachial cells in this lamina.^35,43,50^ Examination of horizontal sections in the mouse revealed a similar population of large lamina I neurons that were coated with VGLUT2^+^ and VGAT^+^ boutons. However, only around one-third of these cells were retrogradely labelled. It is possible that the unlabelled giant cells were not projection neurons, but an alternative explanation is that they project to other brain regions, and this will need to be assessed in future studies.

The large NK1r-immunoreactive cells in laminae III and IV of the rat form another very distinctive and easily recognised population, which was identified in early studies of NK1r distribution.^8,10,25–27,34^ These cells, which respond to noxious stimuli,^36^ were subsequently shown to be projection neurons because >90% could be retrogradely labelled from the caudal ventrolateral medulla and ∼65% from LPb.^52^ In the rat, they receive a highly selective synaptic input from 2 different populations of axons: peptidergic primary afferents, which contain both SP and CGRP,^32^ and NPY-containing axons. The latter are thought to be derived from local inhibitory interneurons and account for one-third of the inhibitory synaptic input.^39,40^ This study shows that cells of this type are also present in the mouse and that they appear to have the same pattern of input from peptidergic primary afferents and NPY-containing interneurons. However, many of them lack the NK1r or express it at low levels. Immunostaining for the receptor cannot therefore be used to reveal this population or to investigate their synaptic inputs, as has been done in the rat.^6,32,33,39^

### 4.3. The neurochemical phenotype of lamina I projection neurons

We previously reported that sst_2A_ expression was restricted to GABAergic neurons in the superficial dorsal horn of both rat and mouse.^37,54^ The finding of sst_2A_ on ∼15% of lamina I neurons retrogradely labelled from the nucleus of the solitary tract in rat^18^ therefore raised the possibility that some projection neurons in this lamina might be GABAergic. Consistent with the findings of Gamboa-Esteves et al.,^18^ we found that ∼10% of lamina I spinoparabrachial neurons were sst_2A_^+^. However, nearly all of these cells also expressed the NK1r, which is thought to be restricted to excitatory (glutamatergic) neurons in this lamina.^25^ We had previously identified a small population of lamina I neurons that expressed both sst_2A_ and NK1r in the mouse,^37^ and the present results suggest that these are all projection neurons. Our finding that 97% of spinoparabrachial axon terminals are VGLUT2-immunoreactive suggests that virtually all spinoparabrachial cells (including those with sst_2A_) are excitatory. Our failure to detect non-GABAergic sst_2A_^+^ neurons^37^ was probably because these constitute a very small population, corresponding to ∼0.5% of all lamina I neurons, compared with 17% of neurons in this lamina that are sst_2A_-immunoreactive.

Although Standaert et al.^48^ detected both enkephalin and dynorphin in lamina I spinoparabrachial neurons, they had used colchicine to increase cytoplasmic peptide levels, and it is now known that this can cause abnormal peptide expression.^13,44^ Our finding that only ∼2% of spinoparabrachial terminals were immunoreactive for enkephalin or dynorphin suggests that few spinoparabrachial neurons normally express significant levels of either of these opioid peptides. However, in agreement with a previous study in the cat,^9^ we found that some spinoparabrachial terminals were SP-immunoreactive. Relatively little is known about SP-expressing neurons in the dorsal horn, as these contain levels of peptide that are not normally detected with immunocytochemistry. However, in situ hybridisation has revealed cells with preprotachykinin 1 mRNA in laminae I and II,^57^ and nonprimary SP-containing axons are seen both here and in the lateral spinal nucleus.^12,51^ As lamina I ALT neurons can generate local axon collaterals in both of these regions,^49^ at least some of the SP-containing nonprimary glutamatergic axons^51^ may be the collaterals of lamina I projection neurons.

## Conflict of interest statement

The authors have no conflicts of interest to declare.

The work was supported by grants from the MRC (MR/L003430/1) and the Wellcome Trust (102645).
